# Bonding Reactions of Dental Self-Adhesive Cements with Synthetic Hydroxyapatite as a Function of the Polymerization Protocol

**DOI:** 10.1155/2021/4572345

**Published:** 2021-11-09

**Authors:** Roger Borges, Carlos Frederico de Oliveira Graeff, Juliana Marchi, Paulo Henrique Perlatti D'Alpino

**Affiliations:** ^1^Centro de Ciências Naturais e Humanas, Universidade Federal do ABC, Santo André, SP, Brazil; ^2^DF-FC, Universidade Estadual Paulista (UNESP), Programa de Pós-Graduação em Ciência e Tecnologia de Materiais (POSMAT), Bauru, SP, Brazil; ^3^Triplet Biotechnology Solutions, São Paulo, SP, Brazil

## Abstract

**Objectives:**

This study evaluated the influence of the cement composition and different polymerization protocols on the bonding chemical interaction of self-adhesive cements with synthetic hydroxyapatite.

**Materials and Methods:**

Two commercial self-adhesive resin cements (RelyX U200 and Maxcem Elite) were selected, manipulated, mixed with hydroxyapatite dry powder (HAp), dispensed into molds, and distributed into three groups according to polymerization protocols: immediate photoactivation (IP); delayed photoactivation, 10 min self-curing and light-curing (DP); and chemical activation (CA, no light exposure). The detailed chemical information, at atomic scale, on the surface and deeper into the bulk of self-adhesive cement/hydroxyapatite mixtures was evaluated with X-ray photoelectron spectroscopy (XPS).

**Results:**

Chemical elements were detected in both cements, such as Na, O, Ca, C, P, and Si. Other elements were detected in minor concentrations. RelyX U200 exhibited the most intense formation of calcium salts products when the cement/HAp mixtures were photoactivated (immediate or delayed). RelyX U200/HAp mixture under delayed photoactivation (DP) also exhibited higher binding energy between calcium moieties of the HAp and methacrylates in the cement. A higher energy difference in the interaction of HAp with the cement comparing the bulk and surface areas was observed when RelyX U200 underwent the delayed photoactivation protocol. Maxcem Elite exhibited an increased chemical reactivity when either chemically activated or immediately photoactivated and a higher binding energy of the carboxyl groups bonded to the calcium of HAp when chemically activated.

**Conclusions:**

The interaction of cements with hydroxyapatite is chemical in nature and leads to the formation of calcium salts, which may favor better integrity and longevity of adhesive restorations. The polymerization protocol affects the chemical interaction in mixtures of self-adhesive cements and hydroxyapatite, influencing the formation of these salts and the establishment of intermolecular interactions between the HAp and the cements.

## 1. Introduction

Self-adhesive resin cements are materials with methacrylate monomers containing phosphoric acid esters that simultaneously demineralize and infiltrate both the smear layer and the underlying dental tissue, forming an interdiffusion zone [1] and providing micromechanical and chemical bonds [2]. The thickness of this interfacial area reaches 2-3 *µ*m or less, depending on the instrument used for preparation [3]. In spite of the insignificant infiltration into the surface of the dental tissues, previous studies using X-ray photoelectron spectroscopy (XPS) have indicated a chemical interaction with calcium from hydroxyapatite, also providing micromechanical retention [4].

The morphology of the interfacial area and the bonding characteristic of self-adhesive cements with dental tissues greatly depends on the functional monomers included in the adhesive formulation, on the reactants formed by the monomers-tooth reaction, and also on the extent to which these monomers interact with the dental substrates [5, 6]. Another characteristic that influences the quality of the interfacial area formed is the fact that the acidity of the newly manipulated self-adhesive cement needs to be neutralized to allow monomer conversion [1]. Deeper in the enamel and dentin, the acidic monomers are gradually neutralized by the mineralized substrate, which tends to lessen their ability to continuously etch these tissues [7]. For that, calcium salts, such as calcium hydroxide, are isolated in the nonacidic part of the formulation, helping to contribute to a more rapid neutralization process [8]. In addition, a glass ionomer concept was incorporated in the formulation of self-adhesive cements, helping to promote a shift towards pH-neutral conditions [9]. The partial surface dissolution of acid-soluble glass present in the composition helps neutralize the cement acidity and release sodium, calcium, silicate, and fluoride ions, which are nearby or take part in the cement setting reaction [8, 10].

The technological advances observed in self-adhesive cements also include a suitable balance between the acidic/hydrophilic monomers, promoting the necessary initial self-adhesion, and the conventional/hydrophobic monomers, also allowing the long-term stability for improved clinical performance of the restoratives [7]. In this manner, considering that the polymerization reaction occurs in an acidic environment, the chemical composition in this category of cement is challenging considering the need of a balanced formula [1, 11]. Thus, it is essential to understand the dynamic processes in the demineralization/monomer permeation and polymerization mechanisms that simultaneously occur at the interfacial area formed between the resin cements and the dental tissue [11].

The present *in vitro* study investigated the bonding chemical interaction between commercial self-adhesive resin cements and synthetic hydroxyapatite. The chemical bonding characteristics of two commercial self-adhesive cements with hydroxyapatite polymerized with different activation protocols were examined using the surface analytical technique XPS. The three main aspects of the bonding reactions analyzed in this work were (i) chemical composition; (ii) formation of calcium salts; (iii) and intermolecular interactions between the self-adhesive cements and hydroxyapatite.

## 2. Materials and Methods

### 2.1. Experimental Design

The factors under study were (1) self-adhesive cements, at two levels: RelyX U200 (3M ESPE, St. Paul, MN, USA) and Maxcem Elite (Kerr Corporation, Orange, CA, USA); (2) polymerization mode at three levels: immediate photoactivation (IP), delayed photoactivation (DP), and chemical activation (CA); and sampling depth of the XPS analysis, at two levels: surface and bulk area. The compositions of the self-adhesive resin cements are described in Table 1. Six experimental groups were categorized according to the proposed polymerization protocols (Table 2).

### 2.2. Specimen Preparation

The specimens were obtained by mixing 0.035 g of dry hydroxyapatite (HAp) with 0.25 g of the self-adhesive cements [11]. The cements were manipulated according to the manufacturers' instructions. Then, the HAp with a high degree of crystallinity and purity was gently incorporated using manual mixing [11]. The mixtures of unpolymerized resin cement/HAp were then placed into a Teflon mold (2 mm thick, 6 mm in diameter), positioned over a polyester strip. After filling the mold, the material surface was covered with a Mylar strip and a glass slide and compressed to extrude excess material. The specimens of groups IP and DP were photoactivated after the working time on the top and bottom surfaces according to the exposure time recommended by the manufacturer. The specimens were then removed from the molds after the photoactivation procedures (IP and DP groups). The light-curing was performed according to the manufacturer's instructions using a polywave LED light-curing unit (Bluephase, Ivoclar Vivadent, Schaan, Liechtenstein) with a radiant emittance of 1200 mW/cm^2^. The light output of the unit was monitored using a handheld radiometer (Model 100, Demetron Research Corp., Danbury, CT, USA). The specimens were stored in lightproof recipients for 24 h at room temperature (22°C). For the chemically activated groups (CA groups), the specimens were obtained in the same way as previously described and then kept in the molds for 24 h at room temperature in the dark prior to their removal. Six replications were made for each test condition (*n* = 6). The results were compared to that of the control group, in which no HA was mixed with the resin cements.

### 2.3. X-Ray Photoelectron Spectroscopy Analysis (XPS)

The bonding reactions of the self-adhesive cements with synthetic hydroxyapatite were characterized using an X-ray photoelectron spectroscopy (K-Alpha X-ray XPS spectrometer, Thermo Scientific, Waltham, MA, USA), in a vacuum of less than 10^−7^ Pa, applying Al-K*α* monochromatic X-ray with a source power of 150 W. The analyses were performed on the surface and in-depth profile (bulk) after etching the specimens to reveal the subsurface information. Both surveys and high-resolution scans were collected. For high-resolution analysis, C1s, Ca2p, and P2p spectra were studied. The survey and high-resolution spectra were analyzed using specific software (Casa XPS, Casa Software Ltd., San Diego, CA, USA). The peak position was calibrated through normalization of the C1s peak at 284.6 eV. A deconvolution approach was applied to the C1s and Ca2p spectra to determine the peak positions and chemical species present in the specimen, as previously analyzed [12, 13]. The chemical elements in the self-adhesive resin cement/HAp mixtures activated using different polymerization protocols were also characterized.

## 3. Results

**Figure 1:**
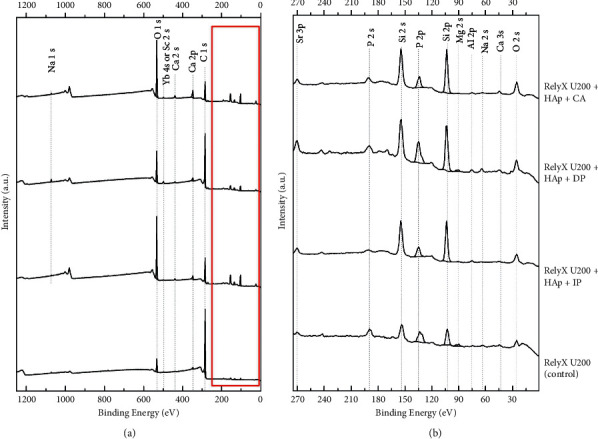
Survey spectra of (from bottom to top) RelyX U200 (control), RelyX U200 + HAp + IP, RelyX U200 + HAp + DP, and RelyX U200 + HAp + CA. On the left (a), survey spectra are presented, while on the right (b) is a zoom-in of the region defined in red.

**Figure 2:**
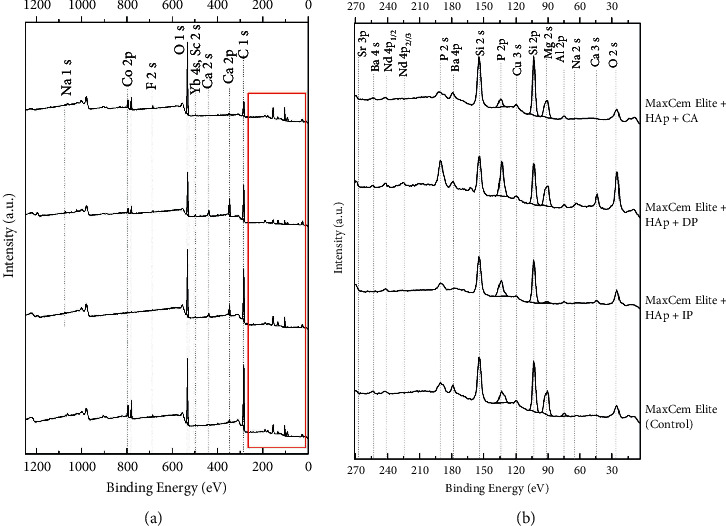
Survey spectra of (from bottom to top) Maxcem Elite (control), Maxcem Elite + HAp + IP, RelyX U200 + HAp + DP, and RelyX U200 + HAp + CA. On the left (a), survey spectra are presented, while on the right (b) is a zoom-in of the region defined in red.

Figures 1 and 2 demonstrate the survey spectra of the cements RelyX U200 and Maxcem Elite, respectively.  Table 3 displays the elemental distribution in atom % observed in the experimental groups. Several chemical elements were detected in both self-adhesive resin cements, such as sodium (Na), oxygen (O), calcium (Ca), carbon (C), phosphorus (P), and silicon (Si). Other elements in minor concentrations were also observed in both cements, such as ytterbium (Yb), strontium (Sr), magnesium (Mg), and aluminum (Al). In the cement from Maxcem Elite, the elements cobalt (Co), fluorine (F), barium (Ba), and neodymium (Nd) were also detected (Table 3).

It can also be observed that the detection of Si is lower when both self-adhesive cements were photoactivated after 10 minutes (Table 3). Magnesium was not detected when the cement RelyX U200 was chemically activated. In addition, a higher detection of F was observed when Maxcem Elite was chemically activated (Table 3). Overall, all these chemical elements are in accordance with those claimed by the manufacturers in their technical profiles.

**Figure 3:**
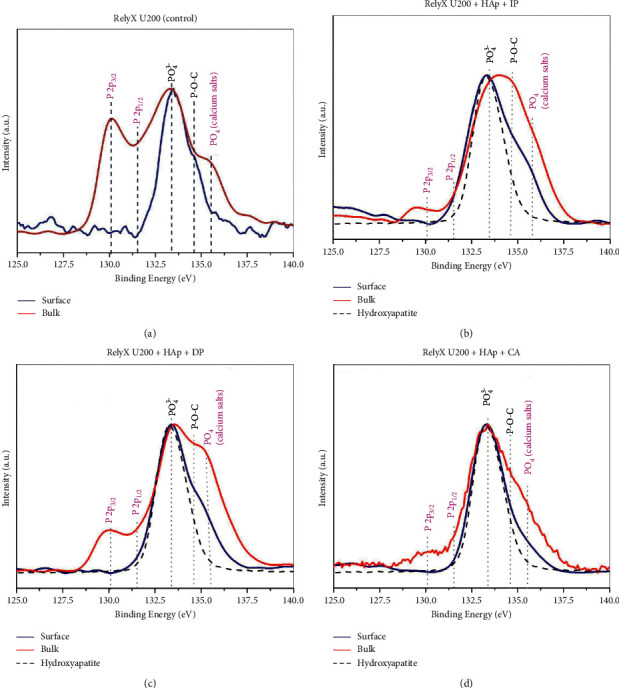
High-resolution P2p spectra of (a) RelyX U200 (control), (b) RelyX U200 + HAp + IP, (c) RelyX U200 + HAp + DP, and (d) RelyX U200 + HAp + CA. The blue lines show the spectra obtained from the surface, while the red spectra were obtained from the bulk. The dashed lines show the spectrum of HAp, which was inserted for comparison purposes. The chemical species assigned to magenta are those that appeared in the spectra, mostly in bulk.

Figure 3 displays the high-resolution P2p spectra of the surface (blue lines) and the bulk (red lines) of RelyX U200 (Figure 3(a)) and of the cement/hydroxyapatite mixtures as a function of the polymerization protocols IP (Figure 3(b)), DP (Figure 3(c)), and CA (Figure 3(d)). For comparative reasons, the P2p spectrum of hydroxyapatite was also displayed (dotted lines). For RelyX U200, the presence of phosphate (PO_4_^3−^, 133.5 eV) and phosphate-oxygen-carbon (P-O-C) bonds (134.5 eV) was observed at the specimen's surface. However, in the bulk area, the presence of other species (assigned in pink), such as phosphate ions related to calcium salts, such as CaHPO_3_ and Ca(H_2_PO_4_)_2_ (PO_4_, ∼135.2 eV), and metallic P-P bonds (P2p_3/2_ and P2p_1/2_, at ∼130.1) was observed. The phosphate species near 133.5 eV are either related to phosphoric acid groups of methacrylate molecules or related to hydroxyapatite particles. The P-O-C groups are possibly related to the phosphoric acid groups of organic molecules. The phosphates from calcium salts and metallic P-P bonds are possible results of the chemical reactions in the setting of self-adhesive cements. Moreover, the groups of RelyX U200 immediately photoactivated (IP, Figure 3(b)) and delayed photoactivation (DP, Figure 3(c)) exhibited the most intense formation of calcium salts products in the bulk area compared with the control (Figure 3(a)) and chemical activation (CA, Figure 3(d)) groups.

**Figure 4:**
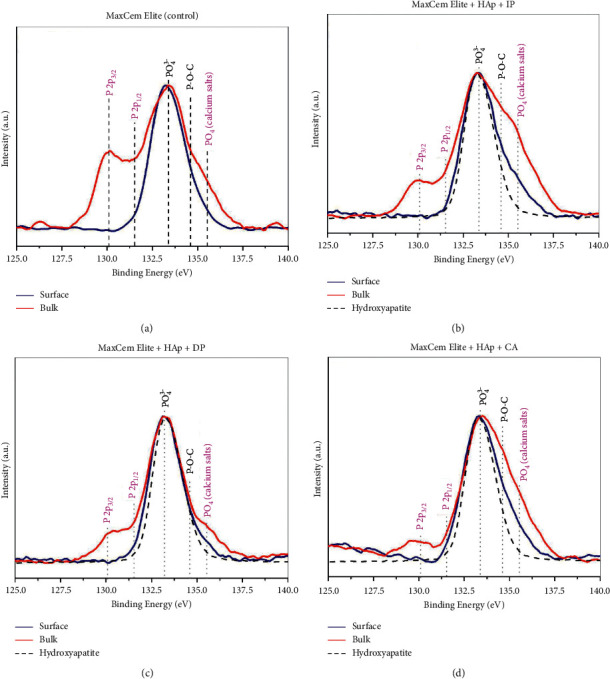
High-resolution P2p spectra of (a) Maxcem Elite (control), (b) Maxcem Elite + HAp + IP, (c) RelyX U200 + HAp + DP, and (d) RelyX U200 + HAp + CA. The blue lines show the spectra obtained from the surface, while the red spectra were obtained from the bulk. The dashed lines show the spectrum of HAp, which was inserted for comparison purposes. The chemical species assigned to magenta are those that appeared in the spectra, mostly in bulk.

In Figure 4, the same comparisons were also displayed with the experimental groups of the cement Maxcem Elite. Similar results were observed, exhibiting richer surface areas of phosphate and P-O-C groups, whereas calcium salts and metallic phosphorous were observed in the bulk area. However, the experimental groups exhibited much less prominent P-O-C bonds than those observed in the cement RelyX U200, irrespective of the activation protocol. Besides, Maxcem Elite (Figure 4(a)) exhibited significantly less P-O-C and calcium salts species when the photoactivation was delayed (DP group) in comparison with other experimental groups, suggesting a diminished chemical reactivity when activated using this protocol. Metallic bonds (P2p_3/2_ and P2p_1/2_) were also observed in the bulk areas of all experimental groups in both resin cements (Figures 3 and 4).

**Figure 5:**
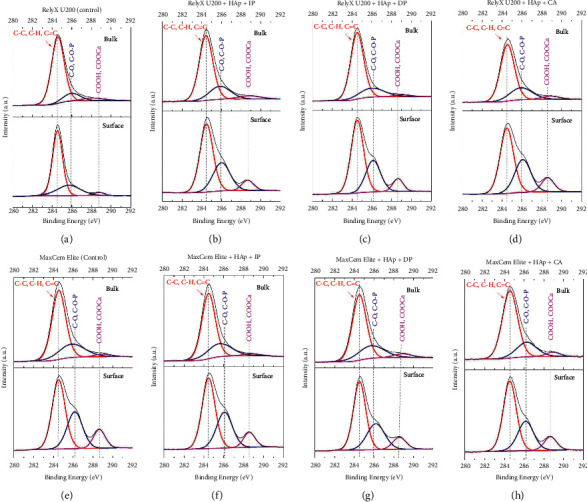
High-resolution C1s spectra of (a) RelyX U200 (control), (b) RelyX U200 + HAp + IP, (c) RelyX U200 + HAp + DP, (d) RelyX U200 + HAp + CA, (e) Maxcem Elite (control), (f) Maxcem Elite + HAp + IP, (g) Maxcem Elite + HAp + DP, and (h) Maxcem Elite + HAp + CA. In each graph, the upper data are related to bulk spectra, while the bottom data are related to surface spectra.

Figure 5 shows the high-resolution C2s deconvoluted spectra of the cement RelyX U200/HAp mixtures polymerized with the protocols IP (Figure 5(b)), DP (Figure 5(c)), and CA (Figure 5(d)), respectively. For comparative reasons, high-resolution C2s deconvoluted spectra of the cement alone (Figure 5(a)) were also displayed. In the same way, the analyses were performed on both the surface and bulk areas of the specimens. Regardless of the experimental group, all specimens showed three prominent peaks: in red, carbon-carbon covalent bond (C-C), carbon-hydrogen bond (C-H), and carbon-carbon double bond (C=C) (284.6 eV); in blue, carbon-oxygen bond (C-O) and carbon-oxygen-phosphate groups (C-O-P) (near 286 eV); and in pink, carboxyl groups (COOH) and calcium carboxylate groups (COOCa) (near 288.5 eV) [12]. By comparing the bulk and surface analyses of all experimental groups, a richer surface was observed in terms of C-O and COOH groups compared to the bulk area.

**Figure 6:**
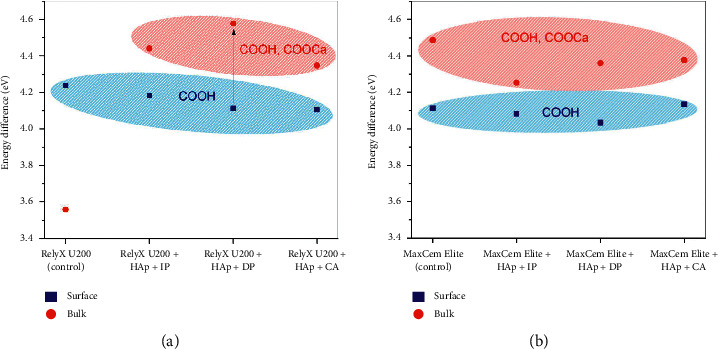
Binding energy difference between the C-H (284.6 eV, a fixed value for adventitious carbon) and COOH (near 286.6) bonds from the high-resolution C1s spectra. (a) Results from the RelyX U200 adhesives experimental groups. (b) Results from the Maxcem Elite adhesives experimental groups. The dots in blue are related to data extracted from the surface analysis, while those in red were extracted from the bulk. The arrow in (a) highlights the higher energy difference between the COOH and COOCa species in the RelyX U200 + HAp + DP experimental group. Similar difference at lower energy was observed for the combination Maxcem Elite + HAp + DP, at lower binding energy. For Maxcem Elite, the highest binding energy of the carboxyl groups bonded to the calcium of HAp (COOCa) was found for the combination when the CA protocol was used.

Figure 6 shows the binding energy differences between the COOH/COOCa and the C-H (284.6 eV) chemical bonds, contrasting the data retrieved from the bulk (red region) and surface (blue region) analysis. The bulk data demonstrated a higher energy difference than that of the surface area, suggesting that the bulk is richer in COOCa bonds considering that these bonds are shifted towards higher binding energy than COOH bonds. Furthermore, a higher energy difference of the interaction of HAp with a cement when the bulk and surface were compared was found when RelyX U200 underwent the delayed photoactivation (DP) protocol (see the arrow in Figure 6(a)), compared with other protocols. Similar difference was observed for the combination Maxcem Elite + HAp + DP, but at lower binding energy (Figure 6(b)). For Maxcem Elite, the highest binding energy of the carboxyl groups bonded to the calcium of HAp (COOCa) was found when the CA protocol was used to activate this cement, compared to other protocols (Figure 6(b)).

**Figure 7:**
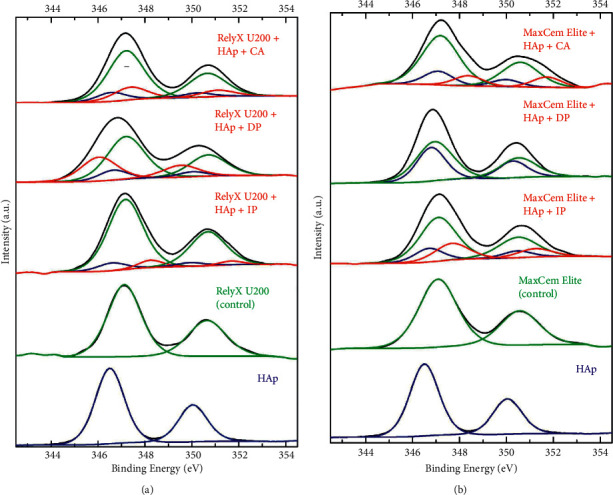
High-resolution Ca2p spectra of (a) RelyX U200 experimental groups and (b) Maxcem Elite experimental groups. The blue spectra are related to HAp, while those in green are associated with the resin cement spectra. The spectra in red appeared during peak deconvolution for improved optimization.

The high-resolution Ca2p spectra of RelyX U200 and Maxcem Elite/hydroxyapatite mixtures are displayed in Figure 7. The deconvoluted spectra of the experimental groups containing HAp show two prominent peaks related to HAp (350.0 and 346.5 eV, blue line) and calcium from the chemical composition of the cements (350.5 and 347.0 eV, green line). These peaks agree with the Ca2p spectra of the HAp and the control groups used in this study, which are also shown in Figure 7. During the deconvolution approach, additional peaks were noticed (red lines). The mixtures RelyX U200/HAp and Maxcem Elite/HAp immediately photoactivated and chemically activated, demonstrated a deconvoluted peak at ∼351.1 and 347.4, which are at similar binding energy than that of control (green line) and HAp (blue line) groups. In contrast, when the photoactivation of the mixture of RelyX U200/HAp was delayed (DP group), deconvoluted peaks at 348.8 and 345.9 eV were observed, which are at a lower binding energy than the control group and the hydroxyapatite group (Figure 7(a)). The experimental group Maxcem Elite/HAp + DP exhibited no extra peaks besides those related to the control and hydroxyapatite references (Figure 7(b)).

## 4. Discussion

Self-adhesive cements are formulated to contain solid inorganic particles and liquid organic compounds, comprising a mixture of monomers and initiators [13]. In the dual curing cement RelyX U200, in addition to conventional methacrylate monomers, it contains methacrylate monomers with phosphoric acid groups with unsaturated carbon-carbon double bonds. These methacrylate phosphoric esters are in the liquid phase of cement mixtures associated with dimethacrylates, acetate, stabilizers, and initiators [13]. In the cement from Maxcem Elite, according to the manufacturer's information, glycerophosphate dimethacrylate (GPDM) is associated with co-monomers (mono-, di-, and trifunctional methacrylate monomers), containing also water, acetone, and ethanol in the liquid phase (Table 1). GPDM is regarded to perform self-etching and bonding to both enamel and dentin [14, 15]. The molecular structure of the acidic monomers is fundamental to obtain strong, aqueous, insoluble, and relatively stable salt complexes with calcium [16]. These phosphoric acidic methacrylates react with the basic fillers and with hydroxyapatite [10]. When in contact with hydroxyapatite, secondary reactions occur by means of chemical bonds [17].

Elements were detected in both self-adhesive resin cements (Figures 1 and 2), mainly demonstrating the chemical compositions of the fillers added to the resin cements. The elemental analysis also revealed a predominance of Si in both self-adhesive cements due to the presence of silica (SiO_2_) in their compositions. For the cement RelyX U200, the peaks of Si (both at 2s and 2p) were higher in the experimental groups when compared to the peaks observed in the control, HAp-free group, irrespective of the activation protocol (Figure 1). Maxcem Elite showed peaks of barium, possibly related to the presence of barium glass fillers and/or barium glass aluminum (Figure 2). Fluoride ion was only found in a detectable amount in the cement Maxcem Elite.

The acidic groups interact with the calcium and phosphate of the hydroxyapatite, with setting reactions involving a complex dynamic mechanism [11]. The main setting reaction occurs via free-radical polymerization, initiated either by curing light or by a redox system, allowing the setting polymerization reaction in this acidic environment [18]. This dynamic setting mechanism, related to the activation mode of the polymerization reaction (chemical or dual) and to the importance of the pH-neutralization, could possibly affect the physicochemical properties of the self-adhesive cements [19]. Acidic monomers, mainly the ones based on phosphates and phosphonates, were developed and added to the formulation of self-adhesive cements to not only demineralize the surface of the dental tissues but also to promote a stable salt formation, mainly associated with calcium [8]. The HAp calcium atoms are crosslinked by organic functional groups, which change their electronic structure [11]. The oxidation state of these calcium atoms is lowered by a charge transfer process with calcium that acts as an electron acceptor [11]. In this manner, the reaction with HAp generates calcium atoms with reduced binding energy, being an electron acceptor [13]. This interaction is most probably based on the chelation of the calcium ions by acid groups and would produce a chemical adhesion to HAp with the tooth structure. These acidic groups bind with the calcium of the HAp, generating monomer-calcium salts, and lead to an increased resistance to hydrolysis and therefore contribute to interfacial bond stability due to the formation of a stabilizing attachment between the methacrylate network and the tooth [16]. Ions released from the acid-soluble filler also neutralize the remaining acidic groups, creating a chelate reinforced three-dimensional methacrylate network [8]. Thus, the interaction of self-adhesive resin cements with hydroxyapatite is chemical in nature and the increased formation of calcium salts may favor better integrity and longevity of adhesive restorations.

Based on the results of the present study, it was found that RelyX U200 exhibited the most intense formation of calcium salt products in the bulk area when immediately photoactivated (IP, Figure 3(b)) and when photoactivation was delayed (DP, Figure 3(c)) when compared with the control group (no associated with HAp) (Figure 3(a)) and with the chemically activated group (CA, Figure 3(d)). RelyX U200 contains phosphoric-acid ester functional monomers that have been associated with improved bond integrity and clinical longevity [20]. Regarding the cement Maxcem Elite, the experimental groups exhibited much less prominent P-O-C bonds than that observed for RelyX U200, regardless of the activation protocol. In addition, Maxcem Elite exhibited significantly less P-O-C and calcium salts species when the photoactivation was delayed (DP group) in comparison with other experimental groups, suggesting comparatively diminished chemical reactivity (Figure 4(a)).

It is worth mentioning that the metallic bonds (P2p_3/2_ and P2p_1/2_) [21] were observed in the bulk area of the experimental groups in both cements (Figures 3 and 4). To the best of our knowledge, no other previous study has identified so far these phosphorous species, and none of the manufacturers mentioned this information in their technical profiles describing the presence of phosphorus allotropes (such as red, black, or white phosphorus).

It has been previously pointed out that the analysis of the C 2s and Ca 2p peaks allowed the investigation of the bonding potential and efficacy of resin-based materials to calcium in HAp [13]. The high surface-to-volume ratio of HAp generates a high amount of reacted HAp, allowing the analysis of the interaction between both substrates and identifies and the possible shifts in the binding energy [5, 13]. Figure 5 displays the results of the high-resolution C 2s deconvoluted spectra of the cement/HAp mixtures in which three main peaks can be observed: C-C, C-H, and C=C (284.6 eV); C-O and C-O-P (near 286 eV); and COOH and COOCa (near 288.5 eV). According to the database, it is not possible to distinguish between C-C and C=C species, as it would be difficult to determine their binding energy. In this case, an independent charging reference would be needed. The peak near 288.5 eV represents an unreacted COOH, which corresponds to the carboxyl groups [5]. The peak near 288.5 eV also results from carboxyl groups bonded to the calcium of HAp (COOCa) [22]. Comparing the bulk and surface results in all experimental groups, a richer surface was observed in terms of C-O and COOH groups compared to the bulk area. Reasons that explain this difference rely on the fact that, deeper in the cement, the HAp is completely involved by the cement in an acidic environment, possibly enhancing the setting reactions and generating more COOCa groups. Conversely, in the surface area, free COOH groups are available to react when in contact with the HAp. It is important to highlight, as previously pointed out, that the self-adhesive cements comprise a hybrid biomaterial in which water-soluble polymerizable monomers were added to the formulation of a conventional glass-polyalkenoate cement, combining their properties [5]. It has been previously reported that these characteristics increase their physicochemical properties and improve the bonding ability to the dental tissues [5, 23].

Regarding the high-resolution C1s spectra, special attention must be given to the peak near 288.5 eV. It provides valuable information about the chemical interaction between carboxyl COOH groups from methacrylate species and calcium ions from the hydroxyapatite powder, forming COOCa bonds [12]. Ionic bonds between the carboxylic groups of polyalkenoic acid, present in the cements and calcium of HAp, produce a significant shift in the carbon C1s peak towards lower binding energy, which indicates the interaction of carboxyl functional groups with HAp [13]. The binding energy differences between the COOH/COOCa and the C-H (284.6 eV) chemical bonds were compared (Figure 6). It was confirmed that the bulk material is richer in COOCa bonds when compared to the surface, in which energy differences were observed. These finds highlight that the HAp found underneath the mixtures' surface is interacting with the methacrylate moieties of the cements. Furthermore, the higher energy difference of the interaction of HAp with a cement when the bulk and surface were compared was found when RelyX U200 underwent the delayed photoactivation (DP) protocol (see the arrow in Figure 6(a)), compared with other protocols. Similar difference in terms of binding energy difference was observed for the combination Maxcem Elite + HAp + DP, but at lower energy (Figure 6(b)). For Maxcem Elite, the highest binding energy of the carboxyl groups bonded to the calcium of HAp (COOCa) was found when the CA protocol was used to activate this cement (Figure 6(b)).

These findings were confirmed by the complementary analysis of the Ca 2p high-resolution spectra (Figure 7). With the analysis of the Ca 2p peak components, a powerful method to quantify the chemical interaction with HAp was developed and adopted to analyze the reaction of composites with inorganic tooth components [13]. These spectra are typically composed of a more intense peak related to Ca2p_3/2_ binding energy and a minor peak related to Ca2p_1/2_ binding energy. Therefore, the deconvoluted data are presented as a pair of peaks. In the present study, the deconvoluted spectra of the experimental groups containing HAp demonstrated two prominent peaks related to hydroxyapatite (350.0 and 346.5 eV, in blue) and calcium present in the chemical composition of the resin cements (350.5 and 347.0 eV, in green). These peaks coincide with those of Ca2p spectra of the hydroxyapatite and the control groups (cements alone) (Figure 7). The peaks assigned in red result from chemical reactions or interactions between the cements and the HAp. In Figures 7(a) and 7(b), the mixtures RelyX U200/HAp and Maxcem Elite/HAp submitted to chemical activation (CA) or immediate photoactivation (IP) showed a pair of peaks (∼351.1 and 347.4 eV) probably related to calcium salts, since several calcium compounds display a Ca2p_3/2_ around 247 eV, including di-calcium phosphate (DCP : Ca_2_P_2_O_7_), tri-calcium phosphate (TCP : Ca_3_(PO_4_)_2_), tetra-calcium phosphate (tetra-CP : Ca_4_P_2_O_9_), calcium phosphate anhydrous (CaHPO_4_), calcium bis(dihydrogen phosphate) (Ca(H_2_PO_4_)_2_), and calcium formate (Ca(HCOO)_2_) [24]. In the RelyX U200/HAp mixture under chemical activation (CA) and immediate photoactivation (IP), 17% and 6% of all calcium present are assigned to calcium salts, respectively. In the MaxElite/HAp mixtures under chemical activation (CA) and immediate photoactivation (IP), calcium salts are 11% and 22% of the total calcium in the specimens, respectively. In contrast, when the mixtures were submitted to delayed photoactivation (DP), different behaviors were observed from those for immediate photoactivation or chemical activation. With RelyX U200/HAp mixture under DP, 30% of all calcium reacted with methacrylate. It is worth mentioning that no calcium salts were observed in this group probably because the peaks related to the cements and those from interactions between the cements and the HAp, which allowed no proper resolution to deconvolute the peaks related to calcium salts. Special attention must be given to the RelyX U200/HAp mixtures considering that a pair of peaks (348.8 and 345.9 eV) was observed, which are likely to be related to calcium ions from the hydroxyapatite interacting with methacrylate moieties of the cements [13, 24]. In this manner, the existence of calcium salts in this group must be considered since the results from high-resolution P2p spectra (Figure 3) showed well-defined, massive PO_4_ moieties related to calcium salts. Interestingly, delaying the photoactivation in the Maxcem Elite/HAp mixtures exhibited no extra peaks, suggesting a poor formation of calcium salts using this protocol, generating no resolved peaks in the deconvolution approach.

Taken together, the results from Figures 6 and 7, it can be inferred that the RelyX U200/HAp mixture under delayed photoactivation (DP group) was the experimental group with the best results regarding the chemical interactions between the cement and the HAp, also suggesting that the DP protocol yielded higher chemical reactivity between the components of the mixtures, highlighted by the presence of calcium salts (Figure 3). For the Maxcem Elite/HAp mixtures, the best result in terms of chemical interactions was the protocol chemical activation (CA group), which also exhibited higher binding energy of the carboxyl groups bonded to the calcium of HAp (COOCa), demonstrated in Figure 7.

The results of the present study were corroborated in a previous study [11] that evaluated different parameters (degree of conversion, free radical entrapment, and chemical interaction) when self-adhesive cement/hydroxyapatite mixtures were polymerized using different activation protocols. It was found that RelyX U200 demonstrated higher dependence on photoactivation (immediate or delayed), whereas Maxcem Elite significantly exhibited significantly higher end conversion when chemically activated [11]. The results of the present study also corroborate with previous studies in which it was found that different polymerization protocols affect the cements in different ways, depending on the parameters tested [25, 26]. It was observed that certain resin cements are more dependent on immediate or delayed photoactivation, whereas others are more dependent on chemical activation [11].

The reason to polymerize the resin cement/HAp mixtures could be argued using delayed photoactivation, a protocol different from that recommended by the manufacturers. Clinically, the immediate photoactivation guarantees the initial stability necessary to withstand clinical tensions [26]. Conversely, when carrying out the photoactivation immediately, the rate of radical propagation becomes limited by diffusion, and the polymerization rate decelerates, allowing a limited conversion, even in the presence of unreacted monomers and free radicals [26]. In addition, there is a fast increase in the cement viscosity induced by light exposure, hampering the reaction of the acidic monomers with the tooth structure, which may affect the bonding mechanism [27]. On the other hand, chemical curing is expected to guarantee the cement to reach its maximum properties over time in areas in which the light energy is unable to reach the material [28]. Conversely, a deficient chemical cure may result in higher concentrations of unreacted double bonds, a lower hardness, and a higher solubility of the cement, which can influence the chemical stability in an oral environment [26]. Yielding a delayed photoactivation, a higher-end conversion could occur [11], allowing improved mechanical properties of the cement [29]. Considering the importance of the bonding mechanism and the need of pH-buffering for polymerization, a time delay between the cement mixing and photoactivation steps may increase the ability of self-adhesive cements to bond to the tooth structures [30]. Although it is not clear if delaying the photoactivation would increase the polymerization potential of resin-based cements [11, 25, 31], it has been found that this protocol reduces the polymerization stress of the resin cements [32, 33]. In this manner, the formulation and different activation protocols of self-adhesive cements seem to synergistically influence the clinical performance of self-adhesive resin cements.

Considering the limitations of the study, the analysis of the binding reactions of self-adhesive cements could be performed on human teeth instead of synthetic hydroxyapatite. Another concern would be the presence of unconsumed, residual acidic monomers in the cement/HAp mixtures that could impact the polymerization reaction of the cement, especially by inhibiting the action of the amine/camphorquinone initiator system of the cements. The presence of residual acidic monomers could occur due to an unbalanced ratio between cement and hydroxyapatite. In spite of these problems, the ratio cement/HAp was in accordance with previously published studies [11, 34]. Additionally, the present study provided crucial complementary information regarding the interaction of this category of cement with dental substrates.

## 5. Conclusions

Within the limitations of this study, the following conclusions can be made:

- Chemical elements found in the analysis are in accordance with those claimed by the manufacturers in the technical profiles
- A higher binding energy is observed in the bulk (a few nanometers underneath the surface) when compared to the surface area, irrespective of the combination cement/activation protocol, demonstrating that the bulk is richer in calcium salts
- Delaying the photoactivation of RelyX U200 for 10 minutes allows the best activation protocol for yielding a higher chemical reactivity between calcium ions from HAp with methacrylate moieties of the cement, highlighted by the presence of calcium salts
- Maxcem Elite presents higher binding energy of the carboxyl groups of the cement bonded to the calcium of HAp and increased calcium salt formation when the chemical activation protocol was used
- Clinical choice of the self-adhesive cement as well as its polymerization protocol can modify its interaction with the crystalline structure of HAp, with possible consequences for the longevity of bonded restorations

## Figures and Tables

**Table 1:** Materials used in this study.

Material	Lot #/expiration date	Composition	Working time (min.)	Setting time (min.)	Exposure Duration (s)	Filler content W (%) V (%)
Maxcem Elite; Kerr Corporation, Orange, CA, USA	6468796 2018-12	GPDM, co-monomers (mono-, di-, and tri-functional methacrylate monomers), water, acetone, and ethanol. Inert minerals and ytterbium fluoride. Automix system.	1.5	4.0	10.0–20.0	69.9 59.0

RelyX U200 3M ESPE, St. Paul, MN, USA	3341399 2019-02	Base: silane-treated glass powder, 2-propenoic acid, 2-methyl-, 1,1'-[1-(hydroxymethyl)-1,2-ethanediyl] ester, reaction products with 2 hydroxy-1,3-propanediyl dimethacrylate and phosphorus oxide, TEGDMA, silane treated silica, sodium persulfate, glass powder, tert-butyl peroxy-3,5,5-trımethylhexanoate. Catalyst: silane treated glass powder, substituted dimethacrylate, 1-benzyl-5-phenyl-barbıc-acid, calcium salt, silane-treated silica, sodium p-toluenesulfinate, 1,12-dodecane dimethacrylate, calcium hydroxide, methacrylated aliphatic amine, methacrylated aliphatic amine, titanium dioxide. Clicker delivery system.	2.0	6.0	20	72.0 43.0

GPDM: glycerol-phosphate dimethacrylate; TEGDMA: triethylene glycol dimethacrylate; UDMA: urethane dimethacrylate; Gly-DMA: glycerol dimethacrylate. All pieces of information were supplied by manufacturers.

**Table 2:** Experimental groups and curing protocols used in the present study.

Polymerization mode used	Group abbreviation	Curing protocol
Immediate photoactivation	IP	Photoactivation only according to the manufacturer's instructions
Delayed photoactivation	DP	10 min delay for chemical curing, followed by photoactivation for 40 s
Chemical activation	CA	Chemical activation only

**Table 3:** Elemental distribution in atom % in the experimental groups as a function of the curing protocols used in the present study.

	Concentration of the elements (atom %)									
	O	Na	Ca	P	C	Si	Mg	N	F	Ba
Hydroxyapatite	51.89 (1.44)	7.13 (0.03)	15.47 (0.65)	16.44 (1.04)	9.08 (1.07)	—	—	—	—	—
RelyX U200	7.90 (0.43)	0.18 (0.06)	0.53 (0.13)	1.65 (0.12)	86.78 (0.71)	2.55 (0.10)	0.43 (0.06)	—	—	—
RelyX U200/HAp IP	35.29 (4.00)	0.17 (0.04)	1.48 (0.03)	4.09 (0.35)	40.50 (6.30)	18.23 (1.94)	0.22 (0.11)	—	—	—
RelyX U200/HAp DP	20.60 (4.13)	0.58 (0.23)	1.25 (0.06)	3.42 (0.28	63.66 (6.24)	9.87 (2.40)	0.63 (0.28)	—	—	—
RelyX U200/HAp CA	42.16 (2.67)	0.28 (0.17)	2.66 (1.05)	3.71 (0.90)	29.04 (4.82)	22.16 (4.26)	—	—	—	—
Maxcem Elite	24.35 (2.42)	—	0.76 (0.06)	2.28 (0.26)	50.66 (1.41)	10.82 (0.71)	9.16 (0.98)	0.57 (0.04)	0.56 (0.01)	0.46 (0.01)
Maxcem Elite/HAp IP	35.04 (8.36)	0.15 (0.04)	2.84 (0.20)	4.74 (0.41)	39.68 (14.33)	16.55 (5.51)	0.71 (0.07)	0.07 (0.10)	0.21 (0.01)	—
Maxcem Elite/HAp DP	25.10 (2.54)	0.23 (0.16)	3.42 (3.75)	3.04 (2.05)	50.51 (5.02)	9.09 (3.09)	6.98 (0.32)	0.85 (0.19)	0.55 (0.06)	0.23 (0.33)
Maxcem Elite/HAp CA	39.00 (0.81)	0.11 (0.05)	0.53 (0.09)	2.44 (0.11)	23.60 (1.91)	19.67 (0.42)	12.48 (0.54)	0.44 (0.00)	1.16 (0.10)	0.59 (0.01)

N = 6

## Data Availability

The data used to support the findings of this study are available from the corresponding author upon request.
